# No-till and nitrogen fertilizer reduction improve nitrogen translocation and productivity of spring wheat (*Triticum aestivum* L.) *via* promotion of plant transpiration

**DOI:** 10.3389/fpls.2022.988211

**Published:** 2022-09-02

**Authors:** Yan Tan, Qiang Chai, Guang Li, Falong Hu, Aizhong Yu, Cai Zhao, Zhilong Fan, Wen Yin, Hong Fan

**Affiliations:** ^1^State Key Laboratory of Aridland Crop Science, Lanzhou, China; ^2^College of Forestry, Gansu Agricultural University, Lanzhou, China; ^3^College of Agronomy, Gansu Agricultural University, Lanzhou, China

**Keywords:** arid region, fertilizer reduction, N accumulation, tillage practice, water use

## Abstract

Excessive nitrogen (N) fertilizer has threatened the survivability and sustainability of agriculture. Improving N productivity is promising to address the above issue. Therefore, the field experiment, which investigated the effect of no-till and N fertilizer reduction on water use and N productivity of spring wheat (*Triticum aestivum* L.), was conducted at Wuwei experimental station in northwestern China. There were two tillage practices (conventional tillage, CT; and no-till with previous plastic film mulching, NT) and three N fertilizer rates (135 kg N ha^–1^, N1; 180 kg N ha^–1^, N2; and 225 kg N ha^–1^, N3). The results showed that NT lowered soil evaporation (SE) by 22.4% while increasing the ratio of transpiration to evapotranspiration (T/ET) by 13.6%, compared with CT. In addition, NT improved the total N accumulation by 11.5% and enhanced N translocation (NT) quantity, rate, and contribution by a range of 6.2–23.3%. Ultimately, NT increased grain yield (GY), N partial factor productivity, and N harvest index by 13.4, 13.1, and 26.0%, respectively. Overall, N1 increased SE (13.6%) but decreased T/ET (6.1%) compared with N3. While, N2 enhanced NT quantity, rate, and contribution by a range of 6.0–15.2%. With the integration of NT, N2 achieved the same level of GY and N harvest index as N3 and promoted N partial factor productivity by 11.7%. The significant positive correlation of NT relative to T/ET and GY indicated that improving T/ET was essential for achieving higher NT. Therefore, we concluded that no-till coupled with N fertilizer rate at 180 kg N ha^–1^ was a preferable management option to boost the N productivity of spring wheat in arid areas.

## Introduction

Currently, agriculture is facing unprecedented global challenges. As food demand by 2050 is expected to increase by at least 60%, nitrogen (N) inputs have grown even greater and faster in recent years than ever before (Vishwakarma et al., 2022). However, the anthropogenic reactive N in the biosphere has already exceeded the proposed planetary boundary (Bjørn et al., 2020), which calls for human beings to stop the continuous utilization of chemical fertilizers. Therefore, it is imperative to explore effective and sustainable ways to improve crop productivity. Overall, enhancing N productivity is promising for addressing the above issues, as N fertilizer recovered from the soil is only 33%, which is far from the expected level (Hawkesford and Griffiths, 2019; Yu et al., 2022).

Generally, N productivity was assessed by N use efficiency, which was divided into three subcomponent traits: N uptake efficiency (NupE, the capacity of plants to take up N from soil), N utilization efficiency (NutE, the ability of plants to convert accumulated N to form grain), and N remobilization efficiency (NreE, the amount of N remobilized to post-anthesis crop components) (Melino et al., 2022). Therefore, enhancing N acquisition, assimilation, and translocation is essential for increasing N productivity (Wu et al., 2021). Numerous studies have been conducted to improve these traits, notably through irrigation management (Li et al., 2018b,^2021^), fertilization management (Chen et al., 2015; Paungfoo-Lonhienne et al., 2019), tillage practices (Yang et al., 2020; Guardia et al., 2021), and cropping systems (Louarn et al., 2021; Quan et al., 2021). Among them, tillage practices in arid areas, especially the no-till, have attracted great attention from politicians, researchers, and farmers due to the increasing threat of water scarcity.

Generally, no-till systems have two contrasting outcomes for N productivity. The majority of studies have declared that no-till could activate the release of soil available N (Yang et al., 2020), promote fertilizer N transformation in soil (Liu et al., 2021), reduce nitrate leaching, and increase plant N acquisition (Giambalvo et al., 2018; Dai et al., 2021). In contrast, others found that no-till enhanced fertilizer N conversion to organic N and fixed NH_4_^+^, causing a decline in crop N uptake (Chen et al., 2021); furthermore, it decreased grain production, resulting in lower NutE (Darapuneni et al., 2019). When we further analyzed the possible reasons for this, we found that cover styles, crop species, and environmental factors that varied among no-till systems led to uncertainty. Therefore, developing studies with specific environmental conditions and typical management strategies are vital to reveal the underlying reason for no-till and thereby diminish uncertainty.

In the Hexi Corridor of northwestern China, a water-limiting area, no-till is widely adopted to conserve soil water (Yin et al., 2021). Wheat (*Triticum aestivum* L.) and maize (*Zea mays* L.) are the two main crop species cultivated in this region. In particular, they were managed in a rotation system where wheat was directly planted on the maize plastic film in the subsequent season. Thus, the wheat received no-till with previous plastic film mulching. It is beneficial for wheat to increase N productivity, as no-till and plastic film mulching can improve soil water status (Yin et al., 2021), and improved water conditions may increase N translocation (NT) (Mon et al., 2016). Moreover, N fertilizer is typically applied to increase N uptake (Tan et al., 2021a). However, whether no-till with previous plastic film mulching will optimize water use to enhance N productivity of wheat is unknown, and whether N fertilization will affect it remains unclear.

In this study, two tillage practices and three N fertilizer rates were utilized, and their effects on water use and N productivity were evaluated. The primary objective of this study was to determine how tillage practices and N fertilization affect water use and N productivity. We hypothesized that no-till with previous plastic film mulching integrated with N fertilizer reduction could improve the N productivity of wheat through (i) improved ratio of transpiration (T) to evapotranspiration (ET), (ii) increased N accumulation, and (iii) enhanced NT. To test this hypothesis, we determined: (i) soil evaporation (SE) and the ratio of T to ET, (ii) N accumulation and translocation, and (iii) grain yield (GY), N partial factor productivity, and the N harvest index.

## Materials and methods

### Experimental site

The field experiment was conducted at the Oasis Agricultural Experimental Station of Gansu Agricultural University (Gansu Province, China; 37°30′N, 103°5′E; 1,776 m a.s.l.) in 2019 and 2020. The station is located in the eastern part of the Hexi Corridor in Northwest China. The long-term (1960–2015) average annual precipitation is lower than 160 mm, whereas the potential evaporation is greater than 2,000 mm. The total rainfall during the wheat growing season in 2020 was lower than that in 2019 (Table 1). The annual temperature is 7.2°C, with an accumulated temperature above 0°C exceeding 3,513°C and above 10°C exceeding 2,985°C, and a frost-free period of 156 days. The soil at the experimental site was classified as an Aridisol (FAO/UNESCO, 1988), with a pH of 8.0 (1:2.5 soil:water), organic carbon of 14.3 g kg^–1^, total N of 0.78 g kg^–1^, NH_4_^+^–N of 1.76 mg kg^–1^, and NO_3_^–^–N of 12.3 mg kg^–1^ prior to the start of the experiment. The soil bulk density in 0–110 cm soil depth averages 1.44 gcm^–3^.

**TABLE 1:** The in-growing-season weather data in 2019 and 2020 at Wuwei Experimental Station, northwestern China.

Year	Item	March	April	May	June	July	Total/average
2019	Mean temperature (°C)	3.3	13.2	15.2	20.2	21.5	14.7
	Relative humidity (%)	31.5	36.7	44.7	56.3	55.8	45.0
	Rain full (mm)	0.0	7.5	26.4	61.3	31.0	126.0
2020	Mean temperature (°C)	4.0	10.7	16.4	21.4	22.6	15.0
	Relative humidity (%)	38.3	25.0	38.0	39.8	52.8	38.8
	Rain full (mm)	1.8	0.0	11.4	10.3	28.4	51.0

### Experimental design and crop management

The experiment followed a split-plot arrangement of treatments in a randomized complete block design with three replicates. The main plot was tillage practice, consisting of conventional tillage (CT) without plastic film mulching and no-till (NT) with previous maize plastic film mulching treatments. The subplot was N fertilizer rate, consisting of 135 kg N ha^–1^ (40% reduction of local N rate, N1), 180 kg N ha^–1^ (20% reduction of local N rate, N2), and 225 kg N ha^–1^ (local N rate, N3). To implement CT, a moldboard plow with a tilling depth of 25–30 cm was used after the maize harvest in the late fall. Then, in the following spring, a tandem disc harrow with a tilling depth of 5–10 cm was applied before wheat sowing. For no-tillage, only a manual duckbill punch roller dibbler was applied at wheat sowing, and the residual plastic film (>70% integrity) of maize was conserved until the next wheat harvest. For N fertilization, urea (46-0-0, N-P-K) with respective N rates of N1, N2, and N3 were broadcast and incorporated into the soil prior to seeding as a base fertilizer. In conjunction with N fertilizer application, all plots received a base application of phosphate fertilizer as calcium superphosphate (0-16-0 of N-P-K) at 100 kg P_2_O_5_ ha^–1^.

Wheat (cv. Long-chun 30) was sown in late March and harvested in late July 2019 and 2020. The row spacing of the wheat population was 15 cm, and the plant density was 4,650,000 plants ha^–1^. The plot size was 10 × 5.5 m^2^. Due to the low precipitation in the testing area (≤160 mm annually), supplemental irrigation was applied. Each plot received 60, 70, and 60 mm of irrigation water at the wheat seedling, booting, and grain-filling stages, respectively. In addition, 120 mm irrigation water in late fall just before soil freezing was applied to all plots as winter storage irrigation. A hydrant pipe system was used for irrigation, and flow meters were used to record the irrigation volume applied to each plot. Between each neighboring plot, a 50-cm wide and 30-cm high ridge was built to eliminate potential water movement.

### Measurement and calculation

#### Soil evaporation

Soil evaporation was determined using microlysimeters (Tan et al., 2021b), which were constructed using polyvinyl chloride tubes with a length of 15 cm, an internal diameter of 11.5 cm, and an external diameter of 12 cm. The base of the tubes was sealed with waterproof tape. Microlysimeters were placed at the center of each plot. Each microlysimeter was filled with *in situ* soil and placed into a larger (12-cm internal diameter) polyvinyl chloride tube that was previously installed in the field. All microlysimeters were weighed at 18:00 at 3–5-day interval from planting to harvest using a portable electronic balance. The SE was recorded and calculated from the weight loss between the two measurements (1-g change was equivalent to 0.1053-mm of SE).

#### Transpiration and ratio of transpiration to evapotranspiration

Evapotranspiration is the sum of T, SE, and canopy evaporation (CE), i.e., the canopy interception rate (Tan et al., 2021a). Commonly, the CE is considered valuable when it meets the following conditions: (i) rain exceeds 5 mm day^–1^ and (ii) the event is followed by five consecutive days (Lawrence et al., 2007). However, these two factors could not be achieved in the testing area. Therefore, the CE was negligible in this study. Accordingly, the consumption of T was calculated as follows:


(1) T=E⁢T-S⁢E

To determine ET, the soil water balance (SWB) method was employed: ET = P + I + *SWF* – *R* – ΔS, where *P* is precipitation during the growing season, *I* is irrigation quota, *SWF* is soil water flux across the lower boundary (110 cm plane), *R* is Runoff, and Δ*S* is the change of soil water storage (SWS) across 0–120 cm soil layer. The *SWF* was quantified based on Darcy’s law for the days of measured soil water content according to Shahadha et al. (2019). Results indicated that the two items were negligible at the experimental site (Yang et al., 2011). Runoff was set to zero due to the low field slope in this study. The ratio of T to ET (T/ET) was created to quantify the proportion of physiological water consumption to total water consumption.

To determine the SWS, the gravimetric water content was first measured. At 0–30 cm depth, the gravimetric water content was measured in 10 cm increments using the oven-drying method, while at 30–120 cm depth, it was measured in 30 cm increments using a neutron probe (CPN 503DR HYDROPROBE^®^, Research Triangle Park, NC, United States) according to Yin et al. (2018). Thereafter, the SWS was determined using the equation *SWS* = θ*v* × *h* × 10, where θ*v* is the volumetric water content at a specific soil layer and *h* is the soil layer depth (cm). The volumetric water content was obtained as the gravimetric water content multiplied by the soil bulk density at each soil layer.

#### Nitrogen accumulation

Plant sampling was conducted 15 days after emergence and at 15-day intervals from the first sampling to the last. For each sampling, a 20 cm length of six rows of wheat plants in each plot was harvested to assess the aboveground dry matter. The leaves, stems, and spikes were dried separately, weighed, and detected. All collected plant samples were oven-dried at 105°C for desiccation and then placed at 80°C until they reached a constant weight. Then, the entire portion of each sample was ground to a fine powder, passed through a 1-mm sieve, milled, and mixed thoroughly for the analysis of N content. A high-induction furnace C and N analyzer (Elementar Vario MACRO cube, Hanau, Hessen, Germany) was used to detect the N content of wheat plant tissues.

The amount of N accumulation (NacA) in wheat plant tissues (kg N ha^–1^) was calculated as the product of the N content and the corresponding dry matter. The rate of N accumulation (NacR) per day in the wheat growing season was calculated as follows:


(2) $$\text{NacR}=\frac{N\,a\,c\,A\,\left(t+1\right)-N\,a\,c\,A\,\left(t\right)}{t\,\left(t+1\right)-t\,\left(t\right)}$$

where NacA is the N accumulation amount (kg N ha^–1^), *i* and *i*+1 are the current and last measuring dates, respectively, and *t* is the number of days after emergence. In this study, t_(i+1)_-t_(i)_ was 15.

#### Nitrogen allocation and translocation

Nitrogen allocation (NA) was defined as the proportion of N accumulated in each plant tissue to total N accumulation and was calculated as follows:


(3) $$\text{NAorgans}=\frac{N\,a\,c\,A\,\text{organs}}{N\,a\,c\,A\,\text{total}}\times 100\%$$

where NA_organ_ and NacA_organ_ represent NA and N accumulation in wheat organs, that is, stems, leaves, and spikes, respectively, and NacA_total_ represents the total amount of N accumulation.

The transformation of accumulated N from vegetative organs (i.e., stems and leaves) to spikes was defined as NT. Thus, the quantity of transferred N (NTQ, kg ha^–1^) was calculated as follows: NTQ = LNacA – FNacA, where LNacA and FNacA are the largest and final amount of N accumulated in stems or leaves; the ratio of transferred N (NTR, %) was calculated as follows: NTR = NTQ/LNacA × 100%; and contribution of transferred N to spike (NTC, %) was calculated as follows: NTC (%) = NTQ/NacA_spike_ × 100%, where NacA_spike_ is N accumulation amount of spike.

In this study, NTC was significantly affected by tillage practices, and the difference in N contribution to the spike between no-till (NT) and CT was defined as the NT advantage (NTA), which was calculated as follows:


(4) $$\text{NTA}=\frac{N\,T\,C_{\text{NT}}}{N\,T\,C_{\text{CT}}}$$

where NTC_NT_ and NTC_CT_ represent the NTC under NT and CT, respectively. Specifically, a value of NTA > 1 indicates an advantage of NT on NT, < 1, indicates a disadvantage, and equal to 1, indicates no difference between NT and CT.

#### Grain yield

Grain yield was assessed for each plot when wheat reached full maturity. After threshing, cleaning, and air drying, the grains were weighed to record the GY.

#### Nitrogen partial factor productivity and nitrogen harvest index

The N partial factor productivity (PFP_N_) is the grain production per unit of N fertilizer applied (Huang et al., 2021) and was calculated as follows:


(5) $$\text{PFPN}=\frac{G\,Y}{N\,f}$$

where Nf is the mineral fertilizer N rate, i.e., 225, 180, and 135 kg N ha^–1^, respectively.

The N harvest index (NHI) was defined as the proportion of N accumulated in grains to the total amount of N accumulation and was calculated as follows:


(6) $$\mathrm{NHI}=\frac{N\,a\,c\,A\,\text{grain}}{N\,a\,c\,A\,\text{total}}$$

where NacA_grain_ represents N accumulation amount of wheat grain.

#### Statistical analysis

The experimental data were analyzed using the statistical analysis software SPSS 17.0 (SPSS Inc., Chicago, IL, United States). Treatment effects were investigated using a split-plot design analysis. Year, tillage practice, and N fertilizer rate were considered fixed effects and replication as random effects. Means were compared using the least significant difference (LSD) test. All determinations of significance were declared at a significance level of 0.05. Linear associations between dependent variables were evaluated using Pearson’s correlation coefficients.

## Results

### Water use characteristics

#### Soil evaporation

Soil evaporation was significantly affected by tillage practice and N fertilizer rate but not by tillage practice × N fertilizer rate interaction and year × treatment interaction (Table 2). On average, SE with no-till and previous plastic film mulching (NT) was reduced by 22.4% compared with CT. There had no significant difference in SE between the N fertilizer rate at 180 kg N ha^–1^ (N2) and 225 kg N ha^–1^ (N3), whereas it with N fertilizer rate at 135 kg N ha^–1^ (N1) was increased by 8.5% compared with N3.

**TABLE 2:** The soil evaporation (SE), transpiration (T), and ratio of transpiration to evapotranspiration (T/ET) of spring wheat during growth period as affected by tillage and N fertilization treatments in 2019 and 2020.

Treatments	SE (mm)		T (mm)		T/ET (%)	
			
	2019	2020	2019	2020	2019	2020
**Tillage effect (T)^a^**						
CT	146.1	131.3	234.3	187.6	61.6	58.9
NT	114.4	101.0	261.3	217.2	69.5	68.2
*P*-value	0.001	0.001	0.026	<0.001	0.003	<0.001
LSD (0.05)	10.5	10.8	21.7	7.4	3.3	1.7
**N fertilization effect (N)^b^**						
N1	134.6	121.2	235.1	193.3	63.6	61.5
N2	130.6	116.8	249.1	201.1	65.6	63.2
N3	125.6	110.4	259.4	212.7	67.4	65.8
*P*-value	0.021	0.015	0.034	<0.001	0.019	0.001
LSD (0.05)	5.6	6.2	16.8	14.3	2.3	2.7
T × N	NS^c^	NS	NS	NS	NS	NS

The effect of year × treatment interaction was omitted due to there were no significant difference.

^a^CT and NT represent conventional tillage without plastic film mulching and no-till with previous plastic film mulching, respectively.

^b^N1, N2, and N3 represent N fertilizer applied at 135, 180, and 225 kg N ha^–1^, respectively.

^c^NS refers to no significant differences between treatments at 0.05 level.

#### Transpiration

Transpiration was significantly affected by tillage practice and N fertilizer rate, but not by tillage practice × N fertilizer rate interaction and year × treatment interaction (Table 2). Compared with CT, T with NT treatment improved by an average of 13.6%. Compared with N3, N1 was reduced by an average of 9.3%. There was no significant difference in T between N2 and N3 in either of the two study years.

#### Ratio of transpiration to evapotranspiration

A significant effect of tillage practice and N fertilizer rate affected the ratio of T to ET (T/ET), but the effect of tillage practice × N fertilizer rate interaction and year × treatment interaction was not significant (Table 2). For the tillage effect, T/ET with NT was enhanced by an average of 14.5% compared with CT. For the N fertilizer effect, T/ET with N1 was reduced by an average of 6.1% compared with N3. There was no significant difference in T/ET between N2 and N3 during the two study years.

### Nitrogen accumulation characteristics

#### Rate of nitrogen accumulation

**FIGURE 1:**
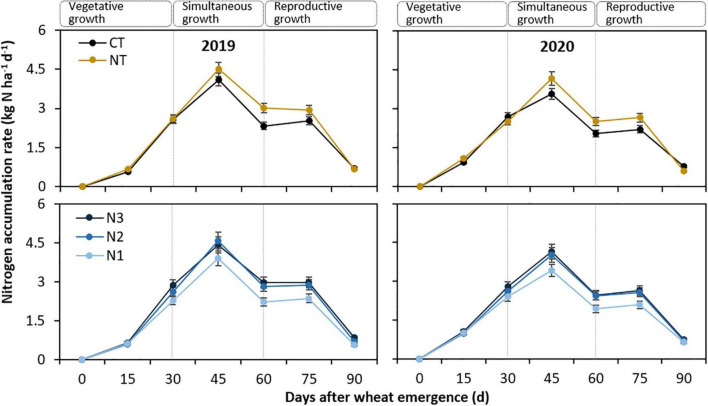
Nitrogen accumulation rate (NacR) of spring wheat with two tillage practices and three N fertilizer rates in 2019 and 2020. CT and NT represent conventional tillage without plastic film mulching and no-till with previous plastic film mulching. N1, N2, and N3 represent N fertilizer applied at 135, 180, and 225 kg N ha^–1^, respectively. The smaller bars are standard error of means (*n* = 3).

The N accumulation rate (NacR) followed a similar curve in different years, but the absolute value differed significantly among the treatments (Figure 1). Since the initial growth, NacR increased until it reached its peak (45 days after emergence), where the values were significantly affected by tillage practice (*P* < 0.001) and N fertilizer rate (*P* = 0.003). For the tillage effect, NacR at peak averaging 4.33 kg N ha^–1^ day^–1^ with NT was improved by 13.1% compared with CT; and averaging 3.66 kg N ha^–1^ day^–1^ with N1 was lowered by 14.9% and 14.6% compared with N2 and N3, respectively. No significant difference in NacR was observed between N2 and N3. Thereafter, NacR decreased until maturity.

Generally, there are three distinct growth stages of wheat: i.e., vegetative, simultaneous, and reproductive (Figure 1 and Table 2). Tillage and N fertilization significantly affected NacR, but the effect varied with the growth stage. During vegetative growth, there were no significant differences in NacR between NT and CT, while that with N1 was lowered by an average of 7.7% and 14.6% compared with N2 and N3, respectively. During simultaneous growth, that with NT averaging 3.54 kg N ha^–1^ day^–1^ was increased by 17.8% compared with CT, and with N1 averaging 2.87 kg N ha^–1^ day^–1^ was lowered by 17.2 and 18.0% compared with N2 and N3, respectively. During reproductive growth, that with NT averaging 1.72 kg N ha^–1^ day^–1^ was increased by 10.3% compared with CT, and with N1 averaging 1.41 kg N ha^–1^ day^–1^ was lowered by 17.3 and 21.7% compared with N2 and N3, respectively.

#### Amount of nitrogen accumulation

**FIGURE 2:**
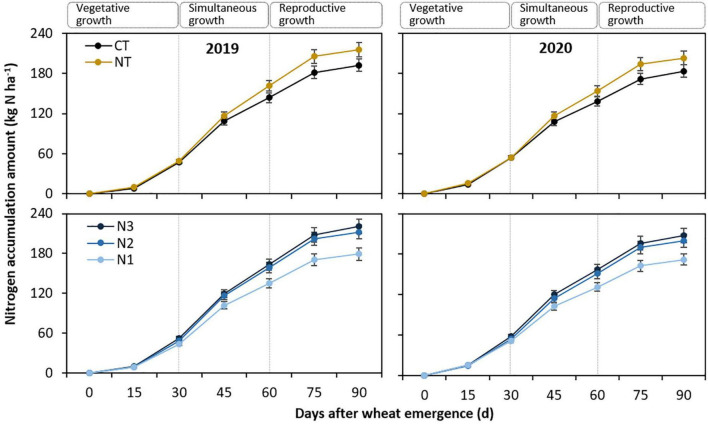
Nitrogen accumulation amount (NacA) of spring wheat with two tillage practices and three N fertilizer rates in 2019 and 2020. CT and NT represent conventional tillage without plastic film mulching and no-till with previous plastic film mulching. N1, N2, and N3 represent N fertilizer applied at 135, 180, and 225 kg N ha^– 1^, respectively. The smaller bars are standard error of means (*n* = 3).

The amount of N accumulation (NacA) followed a similar trend in the two study years (Figure 2, Table 3). In general, simultaneous growth achieved greater NacA (98.3 kg N ha^–1^) than vegetative growth (51.2 kg N ha^–1^) and reproductive growth (49.2 kg N ha^–1^). As NacR was calculated based on NacA (in the “Materials and methods” section), the presentation of treatment difference at the three growth stages for NacA was the same as NacR, even though the absolute values differed greatly from each other. Therefore, this analysis was omitted. The total amount of N accumulation (NacA_total_) was significantly affected by the tillage practice (*P* < 0.001) and N fertilizer rate (*P* < 0.001) but not by tillage practice × N fertilizer rate interaction (*P* = 0.661) and year × treatment interaction (*P* = 0.271). Compared with CT, NacA_total_ with NT averaging 209.4 kg N ha^–1^ was improved by 11.5% (Figure 2). Also, that with N1 averaging 175.5 kg N ha^–1^ was reduced by 14.8 and 18.0% compared with N2 and N3, respectively. There was no significant difference in NacA_total_ between N2 and N3 in either of the two study years.

**TABLE 3:** The N accumulation rate (NacR) and amount (NacA) of spring wheat during 0—0, 30–60, and 60–90 day after emergence as affected by tillage and N fertilization treatments in 2019 and 2020.

Treatments	N accumulation rate (kg N ha^–1^ d^–1^)						N accumulation amount (kg N ha^–1^)					
		
	0–30 d^a^		30–60 d		60–90 d		0–30 d^a^		30–60 d		60–90 d	
						
	2019	2020	2019	2020	2019	2020	2019	2020	2019	2020	2019	2020
**Tillage effect (T)^b^**												
CT	1.57	1.80	3.21	2.81	1.62	1.49	47.2	54.4	96.4	84.2	48.7	44.8
NT	1.63	1.81	3.75	3.33	1.81	1.63	48.9	54.2	112.6	100.0	54.4	48.8
*P*-value	NS^c^	NS	0.005	<0.001	0.27	0.034	NS	NS	<0.001	0.001	0.032	0.026
LSD (0.05)	0.34	0.18	0.42	0.15	0.16	0.10	10.3	5.5	10.1	8.8	3.8	2.0
**N fertilization effect (N)^d^**												
N1	1.45	1.70	3.06	2.68	1.46	1.36	43.5	50.9	91.7	80.3	43.8	40.8
N2	1.60	1.80	3.70	3.23	1.78	1.63	48.0	54.1	111.0	96.9	53.4	48.8
N3	1.75	1.92	3.69	3.30	1.91	1.69	52.6	57.7	110.8	99.1	57.4	50.7
*P*-value	0.001	0.003	0.008	0.001	0.028	0.009	0.002	0.005	0.010	0.002	0.023	0.044
LSD (0.05)	0.11	0.11	0.37	0.24	0.31	0.18	3.2	3.1	12.8	7.7	12.7	7.3
T × N	NS	NS	NS	NS	NS	NS	NS	NS	NS	NS	NS	NS

The effects of year × treatment interaction were omitted due to there were no significant difference.

^a^0–30, 30–60, and 60–90 day after emergence represents the growth stages from emergence to jointing, jointing to flowering, and flowering to maturity.

^b^CT and NT represent conventional tillage without plastic film mulching and no-till with previous plastic film mulching, respectively.

^c^NS refers to no significant differences between treatments at 0.05 level.

^d^N1, N2, and N3 represent N fertilizer applied at 135, 180, and 225 kg N ha^–1^, respectively.

### Nitrogen translocation characteristics

#### Nitrogen allocation

**FIGURE 3:**
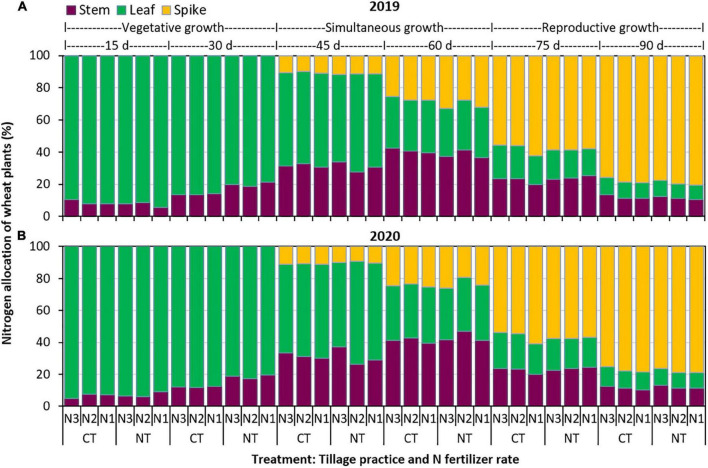
Nitrogen allocation (NA) in the stem, leaf, and spike of wheat plants with two tillage practices and three N fertilizer rates in **(A)** 2019 and **(B)** 2020. CT and NT represent conventional tillage without plastic film mulching and no-till with previous plastic film mulching. N1, N2, and N3 represent N fertilizer applied at 135, 180, and 225 kg N ha^– 1^, respectively.

Leaves were the dominant portion in terms of NA during vegetative growth, while since simultaneous growth, it was gradually replaced by stems and spikes (Figure 3). During reproductive growth, the spike dominated the NA among the three organs. Overall, tillage practice had no significant effect on the NA of stems (*P* ≥ 0.495) and spikes (*P* ≥ 0.353), but that of leaves with NT was significantly (*P* ≤ 0.001) decreased by an average of 10.4 and 11.1% at 75 days and 90 days after emergence, respectively. The N fertilizer rate significantly affected the NA of stems (*P* < 0.001) and leaves (*P* < 0.001) 90 days after emergence but had no significant (*P* = 0.069) effect on the spike. Compared with N3, the NA of stems and leaves at 90 days after emergence with N1 was reduced by 15.7 and 9.5%, respectively, and that with N2 was reduced by 12.1 and 9.3%, respectively. No significant differences in the NA of stems and leaves were found between N1 and N2.

#### Nitrogen translocation

Tillage practice and N fertilizer rate significantly affected the quantity of transferred N (NTQ), ratio of transferred N (NTR), and contribution of transferred to spike (NTC), whereas tillage practice × N fertilizer rate interaction and year × treatment interaction had no significant effect (Table 4). For the tillage effect, the NTQ from stems and leaves with NT increased by an average of 21.8 and 23.3%, respectively, compared with CT. Additionally, the NTR from stems and leaves with NT increased by an average of 6.2 and 6.7% compared with CT, respectively. Similarly, the NTC from stems and leaves with NT increased by an average of 11.9 and 13.5%, respectively, compared with CT.

**TABLE 4:** The quantity of transferred N (NTQ), ratio of transferred N (NTR), and contribution of transferred N to spike (NTC) from stem and leaf as affected by tillage and N fertilization treatments in 2019 and 2020.

Treatments	NTQ (kg ha^–1^)				NTR (%)				NTC (%)			
			
	Stem		Leaf		Stem		Leaf		Stem		Leaf	
						
	2019	2020	2019	2020	2019	2020	2019	2020	2019	2020	2019	2020
**Tillage effect (T)^a^**												
CT	35.4	36.0	52.3	52.6	60.2	61.6	72.5	71.4	23.7	25.5	35.1	37.3
NT	42.4	44.7	65.1	64.2	64.7	64.6	78.0	75.6	27.0	28.2	41.5	40.2
*P*-value	0.013	0.001	0.003	0.011	0.039	0.016	0.002	0.008	0.046	0.022	0.012	0.049
LSD (0.05)	5.6	2.9	6.6	8.8	4.0	2.3	2.6	2.8	2.8	2.5	5.0	2.8
**N fertilization effect (N)^b^**												
N1	33.0	35.5	53.0	55.9	63.7	65.3	76.6	75.5	24.5	26.1	39.3	41.2
N2	44.3	46.2	63.9	63.0	65.0	66.7	76.4	74.9	27.4	29.3	39.5	40.0
N3	39.4	39.4	59.1	56.2	58.5	59.5	72.7	70.1	24.1	25.1	36.2	35.8
*P*-value	0.001	0.002	0.010	0.039	0.004	<0.001	0.008	0.001	0.042	0.014	0.041	0.035
LSD (0.05)	3.8	3.5	4.7	6.1	3.1	2.0	2.2	2.0	2.6	2.5	3.2	3.8
T × N	NS^c^	NS	NS	NS	NS	NS	NS	NS	NS	NS	NS	NS

The effects of year × treatment interaction were omitted due to there were no significant difference.

^a^CT and NT represent conventional tillage without plastic film mulching and no-tillage with previous plastic film mulching, respectively.

^b^N1, N2, and N3 represent N fertilizer applied at 135, 180, and 225 kg N ha^–1^, respectively.

^c^NS refers to no significant differences between treatments at 0.05 level.

**FIGURE 4:**
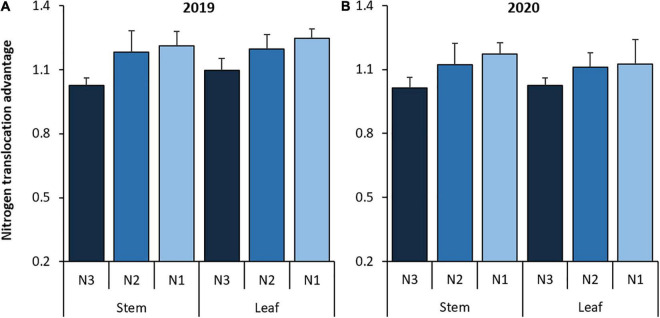
Nitrogen translocation advantage (NTA) of stem and leaf with NT relative to CT during the wheat growing season in **(A)** 2019 and **(B)** 2020. CT and NT represent conventional tillage without plastic film mulching and no-till with previous plastic film mulching. N1, N2, and N3 represent N fertilizer applied at 135, 180, and 225 kg N ha^– 1^, respectively. The smaller bars are standard error of means (*n* = 3).

For the N fertilizer effect, N2 consistently had higher NTQ, NTR, and NTC than N3. On average, NTQ from stems and leaves with N2 increased by 14.9 and 10.1%, respectively; NTR from stems and leaves with N2 increased by 11.7 and 6.0 %, respectively, and NTC from stems and leaves with N2 increased by 15.2 and 10.4 %, respectively, compared with N3 (Table 4). In addition, the NTA from stems and leaves with N2 increased by 12.9 and 8.6%, respectively, compared with N3 (Figure 4). Further lowering of the N fertilizer rate also increased NTA. Compared with N3, NTA from stems and leaves with N1 increased by 16.9 and 11.7%, respectively. However, in most cases, the differences in NTR, NTC, and NTA in both stems and leaves between N1 and N2 were not significant.

### Yield response

Grain yield was significantly affected by tillage practice, N fertilizer rate, and tillage practice × N fertilizer rate interaction but not by year × treatment interaction (Table 4). For the CT treatments, the GY with N1 decreased by an average of 8.1 and 16.8% compared with N2 and N3, respectively. In addition, N2 decreased by an average of 9.4% compared with N3. For the NT treatments, N1 decreased by an average of 16.3 and 18.5% compared with N2 and N3, respectively. However, there was no significant difference in GY between N2 and N3. Overall, GY with NT was enhanced by 13.4% compared with that with CT.

### Nitrogen partial factor productivity

The tillage practice, N fertilizer rate, and tillage practice × N fertilizer rate interaction significantly affected N partial factor productivity (PFP_N_), but the year × treatment interaction PFP_N_ did not (Table 5). For the CT treatments, PFP_N_ with N1 was enhanced by an average of 13.2 and 38.7% compared with N2 and N3, respectively. In addition, N2 was enhanced by an average of 22.6% compared with N3. For the NT treatments, N1 was enhanced by an average of 21.7 and 35.8% compared with N2 and N3, respectively. In addition, N2 was enhanced by an average of 11.7% compared with N3. Overall, PFP_N_ with NT was enhanced by 13.1% compared with that with CT.

**TABLE 5:** The grain yield (GY), N partial factor productivity (PFP_N_), and N harvest index (NHI) of spring wheat as affected by tillage and N fertilization treatments in 2019 and 2020.

Tillage practice^a^	N fertilizer rate^b^	GY (t ha^–1^)		PFP_N_		NHI	

		2019	2020	2019	2020	2019	2020
CT	N1	5.78	5.87	42.8	43.5	0.64	0.56
	N2	6.52	6.17	36.2	34.3	0.55	0.48
	N3	7.10	6.91	31.5	30.7	0.57	0.50
NT	N1	6.66	6.06	49.3	44.9	0.70	0.68
	N2	8.07	7.13	44.8	39.6	0.74	0.64
	N3	7.83	7.77	34.8	34.5	0.69	0.69
LSD (0.05)		0.24	0.30	1.4	1.9	0.040	0.039
***P*** **-value^c^**							
Tillage practice (T)		<0.001		<0.001		0.001	
N fertilizer rate (N)		<0.001		<0.001		0.012	
T × N		0.002		0.007		0.006	

The effects of year × treatment interaction were omitted due to there were no significant difference.

^a^CT and NT represent conventional tillage without plastic film mulching and no-tillage with previous plastic film mulching, respectively.

^b^N1, N2, and N3 represent N fertilizer applied at 135, 180, and 225 kg N ha^–1^, respectively.

^c^The P values with each indicator were for the two study years.

#### Nitrogen harvest index

The N harvest index (NHI) of wheat was significantly affected by tillage practice, N fertilizer rate, and tillage practice × N fertilizer rate interaction but not by the year × treatment interaction (Table 5). For CT treatments, NHI with N1 was improved by an average of 12.0 and 16.6% compared with N2 and N3, respectively. No significant difference in NHI was found between N2 and N3 patients. For NT treatments, there was no significant difference in NHI among the three N fertilizer treatments. Overall, NHI with NT was enhanced by an average of 26% compared with CT.

### Relationship of nitrogen translocation relative to water use, grain yield, and nitrogen use

**FIGURE 5:**
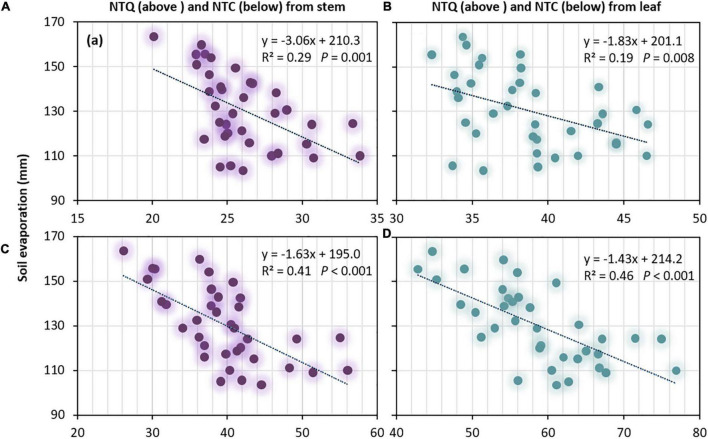
Soil evaporation (SE) followed in a negative linear regression curve with the quantity of transferred N (NTQ) from **(A)** stem and **(B)** leaf and with a contribution of transferred N (NTC) from **(C)** stem and **(D)** leaf of spring wheat across 2019–2020.

**FIGURE 6:**
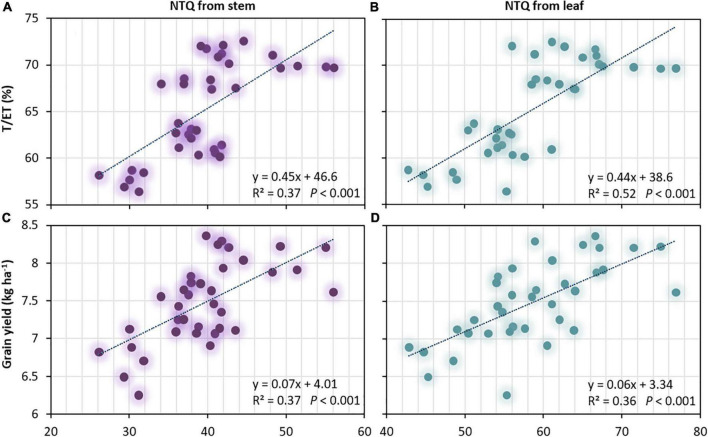
Ratio of transpiration to evapotranspiration (T/ET) and grain yield (GY) both followed in a positive linear regression curve with the quantity of transferred N (NTQ) from **(A,C)** stem and **(B,D)** leaf of spring wheat across 2019–2020, respectively.

The significant negative linear regression of SE relative to NTQ, NTR, and NTC indicated that higher SE greatly restricted the NT of wheat from both stems and leaves (Figure 5 and Table 6). A significant positive linear regression of T/ET relative to NTQ from stems and leaves, and of GY relative to NTQ from stems and leaves were revealed, implying that improving T/ET will enhance NT (as water conservation due to lowered SE will improve NTQ), and enhancing NT can boost grain production (Figure 6). However, the correlation between GY, NTR, and NTC was not significant (Table 6). Moreover, T, PFP_N_, and NHI were more related to NTQ, NTR, and NTC in leaves, implying that the improved leaf NT due to higher T contributed more to the promotion of N harvest in wheat grains.

**TABLE 6:** Pearson’s correlation coefficients (*df* = 36) of N translocation quantity (NTQ), ratio (NTR), and contribution (NTC) of stem and leaf relative to soil evaporation (SE), transpiration (T), ratio of transpiration to evapotranspiration (T/ET), grain yield (GY), N partial factor productivity (PFP_N_) and N harvest index (NHI) of spring wheat.

Variable^a^	SE	T	T/ET	GY	PFP_N_	NHI
NTQs	−0.629**^b^	0.120	0.612**	0.610**	0.248	0.321
NTQl	−0.668**	0.351*	0.718**	0.614**	0.641**	0.558**
NTRs	−0.343*^b^	0.133	0.121	0.101	0.281	0.235
NTRl	−0.355*	0.371*	0.448**	0.132	0.775**	0.690**
NTCs	−0.537**	0.288	0.360*	0.139	0.252	0.259
NTCl	−0.448**	0.435*	0.337*	0.258	0.626**	0.517**

^a^NTQs and NTQl represent N translocation quantity from stem and leaf, NTRs and NTRl represent N translocation ratio from stem and leaf, and NTCs and NTCl represent N translocation contribution of stem and leaf, respectively.

^b^**Correlation coefficient was significant at P < 0.01.

*Correlation coefficient was significant at P < 0.05.

## Discussion

### Optimization of water use

Soil evaporation is nonproductive water use (Tugwell-Wootton et al., 2020), which constitutes up to 40% of ET in terrestrial environments (Balwinder et al., 2011). Therefore, suppressing evaporation is vital for improving T and enhancing crop productivity (Balwinder et al., 2011). In general, soil evaporation is jointly influenced by meteorological status (e.g., air temperature, solar radiation, wind speed, and air humidity) and edaphic conditions (e.g., soil temperature, soil water content, moisture availability, and soil porosity) (Zribi et al., 2015; Liao et al., 2021). As meteorological conditions are hardly influenced, the edaphic conditions moderated by agronomic measures become the crux for depressing soil evaporation.

Tillage practices with variable mulching options are favorable for the optimization of edaphic conditions (Naveen et al., 2021); therefore, they have been widely proposed to conserve soil water (Zhang et al., 2022). In this study, wheat uniquely received no-till with plastic film mulching (NT) from previous maize, which lowered soil evaporation during the wheat-growing season. On average, soil evaporation with NT was reduced by 22.4% compared with CT. This was mainly attributable to the presence of plastic films and maize residues. The mulching of plastic film forms physical isolation at the soil surface, which slows down and hinders the exchange of water between the soil and air (Li et al., 2018a; Liao et al., 2021). Maize residues intercept solar radiation and reduce wind speed close to the soil surface, which lowers water movement from the soil to the atmosphere. Thus, more water was preserved with NT than under evaporation to support physiological water use. Overall, NT with T averaging 239.2 mm was improved by 13.6%, and the ratio of T to ET (T/ET) averaging 68.9% was enhanced by 14.5% compared with CT.

Nitrogen fertilization is particularly applied to increase the T of field crops, while also reducing soil evaporation (Ogola et al., 2002; Tan et al., 2021b), as it could enhance the development of crop canopy and improve canopy shading on the land surface, thus lowering the soil temperature (Zhou et al., 2019). In this study, reducing the N fertilizer rate was found to be instructive for increasing soil evaporation. Compared with the local N rate (N3), the treatment with a 40% reduction in the local N rate (N1) with soil evaporation averaging 127.9 mm increased by 8.5%. However, the treatment with a 20% reduction in the local N rate (N2) showed no significant difference in soil evaporation with N3, indicating that lowering the N rate by 20% barely affected wheat canopy development (i.e., growth status). Thus, the local N rate might be excessively applied, resulting in damage to the environment owing to heavy N losses (Liu et al., 2022). Furthermore, N1 treatment led to a 9.3% reduction in T and a 6.1% reduction in T/ET compared with N3, whereas no significant difference in T and T/ET was found between N2 and N3. This illustrates that a 20% reduction in the N rate achieved an equal capacity for water usage, which is preferable for wheat production. However, this study did not evaluate environmental effects such as greenhouse gas emissions, ammonia volatilization, and nitrate leaching, which partially supported our conclusion. Therefore, further research is required to comprehensively evaluate this effect.

### Enhancement of nitrogen translocation

During the reproductive growth of field crops, growing grains represent a strong sink for resources that require a large amount of physiologically available N (Bertheloot and Andrieu, 2008). However, N absorption by plants during this period is slow (Xu et al., 2005). This was confirmed in this study, as the N accumulation rate and amount were all declined during reproductive growth. This is mainly due to plant senescence, especially in the roots and leaves (Li et al., 2019). Nevertheless, adopting suitable tillage practices could increase N uptake; that is, N accumulation, as more soil available N is provided (Zhang et al., 2020; Pecci Canisares et al., 2021). This was also evidenced by our research, wherein employing NT led to an increase of 10.3% in the N accumulation rate and amount (same value for rate and amount due to the calculation method) compared with CT during reproductive growth. In addition, they were increased by 17.8% during simultaneous growth (i.e., vegetative and reproductive growth simultaneously progressed). Accordingly, the total amount of N accumulation with NT was improved by 11.5% compared with that with CT. Improved water uptake due to the adoption of conservation tillage was also attributable to improved N accumulation (Chai et al., 2022).

Numerous studies have demonstrated that N fertilizer application can increase plant N accumulation, whereas excessive N supply leads to soil, water, and air damage (Chen et al., 2014). Improved synchronization of the N supply and requirements according to crop growth features is fundamental for N application management (Hu et al., 2020). In this study, the N supply for wheat was reduced by 20 and 40% based on local N rarity, while the two options had contrasting effects on N accumulation. Generally, the N accumulation rate and amount of wheat at the three growth stages with N1 were all lower than those of N2 and N3, but there was no significant difference between N2 and N3. Specifically, the N accumulation rate and amount in N1 were reduced by 7.7 and 14.6% during vegetative growth, 17.2 and 18.0% during simultaneous growth, and 17.3 and 21.7% during reproductive growth, respectively, compared with N2 and N3. This directly led to the total amount of N accumulation, with N1 being reduced by 14.8 and 18.0%, respectively, compared with N2 and N3. Therefore, a 40% reduction in N fertilizer generated an increase in soil available N (Hu et al., 2018), which is unsuitable for wheat production. N fertilizer reduction at 20% could achieve the same capability of N accumulation, which can be attributed to increased soil N stocks and optimized interactive nutrient effects on N availability (Aulakh and Malhi, 2004; Ladha et al., 2005).

As N absorption is limited during reproductive growth, the transfer of N from vegetative organs (i.e., leaves and stems) to grains is of vital importance (Xu et al., 2005). Generally, tillage practices influence the N metabolism of field crops (Wang et al., 2015), which potentially affects NT. In this study, increased NT with the adoption of NT was observed, even though NA in plant organs was slightly affected. Specifically, the NA for leaves under NT declined by 11.1% at maturity, but that for stems and spikes was not significantly influenced. For NT, the quantity of transferred N (NTQ) from stems and leaves, ratio of transferred N (NTR) from stems and leaves, and contribution of transferred N (NTC) from stems and leaves increased by 21.8 and 23.3%, 6.2 and 6.7%, and 11.9 and 13.5% compared with CT, respectively. The primary reason may be that adopting NT improved the water status and N accumulation, which allowed N to be efficiently and sufficiently transported from vegetative organs to reproductive organs (Ma et al., 2014). Furthermore, no-till can promote the photosynthetic capacity of leaves (Yang et al., 2021), which leads to additional N transportation. In contrast, the NT treatment in this study was quite distinct from the others, as it combined with plastic film mulching, which may accelerate wheat growth and leaf senescence (Gan et al., 2013). Therefore, the procedural promotion of N transport from vegetative organs to grains is activated.

Lowering the N fertilizer rate reduced the N stock in vegetative organs but stimulated N transportation (Ogawa et al., 2016). In this study, the NA at wheat maturity of stems and leaves with N1 was reduced by 15.7 and 9.5%, respectively, and with N2 was reduced by 12.1 and 9.3%, respectively, compared with N3. This shows an obvious NT. However, owing to insufficient N accumulation with N1, the NT performance of N1 against N3 was inconsistent across the years. N2 treatment consistently increased NTQ from stems and leaves by 14.9 and 10.1%, NTR from stems and leaves by 11.7 and 6.0%, and NTC from stems and leaves by 15.2 and 10.4%, respectively, compared with N3. Accordingly, the NTA from stems and leaves with N2 increased by 12.9% and 8.6%, respectively, compared with N3. This implies that N deficiency in the soil accelerated the effect of tillage on wheat NT. However, N fertilizer management should not depend solely on NT. Excessive N reduction inevitably restricts essential N accumulation in plants, leading to an undesirable decrease in crop productivity (Chen et al., 2015; Hu et al., 2018).

### Promotion of the productivity

Grains are the most active sink for N assimilation (Zhong et al., 2019), and NT is a prerequisite for quality (Chen et al., 2020). However, higher NT inconsistently promoted GY. In this study, N2 treatment significantly improved NT, but GY was reduced by 9.4% compared with N3 under the CT treatment, even though a significant positive relationship between GY and NT was revealed (Figure 6 and Table 6). Primarily, the increased soil evaporation with lowered T/ET was attributed to the yield reduction of N2 under CT. NT was negatively correlated with soil evaporation (Figure 5 and Table 6) and positively correlated with T/ET (Figure 6 and Table 6). Unfortunately, N fertilizer reduction consistently increased soil evaporation and decreased T/ET, especially under the CT treatment (data not shown). Nevertheless, the disadvantage of T/ET owing to N fertilizer reduction can be offset by adopting no-till with plastic film mulching. No significant difference in GY between N2 and N3 was found under the NT treatment in either of the two study years. Therefore, in wheat production, reducing the N fertilizer rate with no-tillage practices proved to be the best combination, and T/ET must be considered for improving GY through NT.

Adopting soil and fertilizer management practices can maximize crop N uptake, optimize indigenous soil N supply, and ultimately increase crop yield (Paungfoo-Lonhienne et al., 2019; Guardia et al., 2021). This directly improves the N partial factor productivity and indirectly improves the N harvest index (Hu et al., 2018; Huang et al., 2021). However, in this study, the N1 treatment, with the lowest GY, achieved the highest N partial factor productivity and harvest index. This is mainly because it has the lowest N fertilizer rate, which easily promotes N-related productivity (Hu et al., 2018). However, such a promotion was limited, as fundamental N accumulation declined, which further restricted NT (Chen et al., 2015). Therefore, with N1 treatment with N partial factor, productivity improved by 13.2 and 38.7%, and with N harvest index, it improved by 12.0 and 16.6%, respectively, but the GY decreased by 8.1 and 16.8%, respectively, compared with N2 and N3 under the CT treatment. N2 achieved the triple goal of promoting GY, N partial factor productivity, and N harvest index under NT due to improved NT. This was also verified by the positive correlation of N partial factor productivity and harvest index relative to NT, especially from the leaves (Table 6). Accordingly, reducing the N fertilizer rate to 20% was the most appropriate option in terms of adopting no-till with previous plastic film mulching to boost wheat N productivity. However, this study omitted zero N control when carrying out the field experiment, which led to a lack of determination of apparent N recovery, an important indicator for evaluating the N productivity of this cropping system.

## Conclusion

A previous plastic film with no-till optimized in-season water use and promoted N productivity in spring wheat. Compared with CT, no-till with previous plastic film mulching lowered soil evaporation by 22.4% and increased T/ET by 13.6%; thus, increasing total N accumulation by 11.5% and NT from leaves and stems by 6.2–23.3%. Ultimately, it increased GY by 13.4%, N partial factor productivity by 13.1%, and N harvest index by 26%. Reducing the N fertilizer rate to 20% barely affected the water capture capability of wheat, but greatly improved NT. As a result, the NTA of no-till relative to CT from stems and leaves increased by 12.9 and 8.6%, respectively, compared with N3. Accordingly, a 20% reduction in the N fertilizer rate scarcely lowered GY, as there was a significant positive correlation between GY and NT. In addition, NT was positively correlated with T/ET. Therefore, no-till with previous plastic film mulching and N fertilizer reduction at 20% was found to be the preferable agronomic management for sustainable wheat production in arid areas.

## Data availability statement

The raw data supporting the conclusions of this article will be made available by the authors, without undue reservation.

## Author contributions

CZ, AY, and ZF: data curation. YT: formal analysis and writing—original draft. WY and FH: methodology. YT and FH: resources. HF: software. FH: validation. QC and GL: writing—review and editing. All authors approved the final version of the manuscript.
